# Outcomes among patients with hepatorenal syndrome based on hospital teaching and transplant status: Analysis of 159 845 hospitalizations

**DOI:** 10.1002/jgh3.12985

**Published:** 2023-11-27

**Authors:** Aalam Sohal, Hunza Chaudhry, Dino Dukovic, Kris V. Kowdley

**Affiliations:** ^1^ Department of Hepatology Liver Institute Northwest Seattle Washington USA; ^2^ Department of Internal Medicine University of California Fresno California USA; ^3^ Department of Medicine Ross University School of Medicine Miramar Florida USA; ^4^ Department of Medicine, Elson S. Floyd College of Medicine Washington State University Pullman Washington USA

**Keywords:** hepatorenal syndrome, national inpatient sample, outcomes, teaching status, transplant status

## Abstract

**Background and Aim:**

Hepatorenal syndrome (HRS) is a life‐threatening complication of advanced liver disease. This study aimed to examine the impact of hospital teaching/transplant status and availability of liver transplantation on survival among hospitalized patients with HRS in the United States.

**Methods:**

Patients with HRS were identified from the national inpatient sample 2016–2019. Information was collected regarding patient demographics, hospital characteristics, liver disease etiology, presence of liver disease decompensations, Elixhauser comorbidities, and interventions. Patients were classified as being treated at three hospital groups: non‐teaching hospitals (NTHs), teaching non‐transplant centers (TNTCs), and teaching transplant centers (TTCs). The relationship between hospital teaching/transplant status and in‐hospital mortality and transplant‐free mortality was examined using multivariable linear and logistic regression analysis.

**Results:**

A total of 159,845 patients met the criteria for HRS. Of these, 24% were admitted to NTHs, 50.8% to TNTCs, and 25.2% to TTCs. Admission to a TTC was independently associated with a lower mortality risk compared to admission to non‐TTCs (aOR = 0.75, 95% CI: 0.68–0.83, *P* <0.001). Patients at TTCs had a lower transplant‐free mortality risk than those at NTHs (aOR = 0.75, 95% CI: 0.67–0.83, *P* < 0.001). There was no significant difference in all‐cause or transplant‐free mortality between TNTCs and NTHs.

**Conclusion:**

Patients with HRS admitted to TTCs have higher disease severity, but significantly improved outcomes compared to those admitted to NTHs. These data suggest opportunities for increased disease awareness and education among NTHs and support early referral for liver transplant evaluation among hospitalized patients with HRS.

## Graphical abstract

Study shows that patients with hepatorenal syndrome admitted to teaching transplant centers have better outcomes compared to patients admitted to teaching non‐transplant centers and non‐teaching centers, regardless of whether they receive the transplant or not.

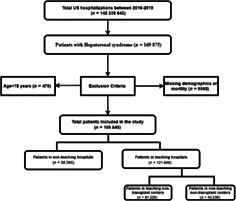

## Introduction

Hepatorenal syndrome (HRS), a severe complication of cirrhosis, develops as a result of alteration in hepatic and systemic hemodynamics due to splanchnic vasodilation and reduced systemic vascular resistance.^1^ Previously, HRS was classified as rapidly progressive (HRS type 1) and slowly progressive (HRS type 2).^2^, ^3^, ^4^ In 2015, HRS‐1 was reclassified as hepatorenal syndrome with acute kidney injury (HRS‐AKI) and HRS‐2 as HRS‐non‐AKI (HRS‐NAKI) by the international club of ascites (ICA).^4^ HRS‐AKI has an estimated annual incidence ranging from 9000 to 35 000 patients and has been consistently demonstrated to have high morbidity, mortality, and resource utilization.^5^, ^6^, ^7^, ^8^, ^9^, ^10^, ^11^ A recent study reported a 50% reduction in‐hospital mortality between 2005 and 2014, likely due to increased utilization of liver transplantation (LT) and renal replacement therapy (RRT).^7^

For patients with HRS‐AKI, the only treatment shown to improve long‐term survival is LT.^12^ Terlipressin has long been used for the management of hepatorenal syndrome in several countries and was recently approved by the FDA for use in the United States.^13^ Prior to the approval of terlipressin in the United States, the management of patients with HRS was limited to the “off‐label” use of vasopressors such as norepinephrine in the ICU setting and midodrine/octreotide in lower acuity hospital settings.^12^, ^14^, ^15^ Vasoconstrictors have been shown to improve short‐term survival in patients with HRS but are associated with an increased risk of complications.^13^, ^14^, ^15^, ^16^, ^17^, ^18^, ^19^

Terlipressin, recently approved by the FDA, was effective when used in appropriate patients with HRS.^20^, ^21^ A meta‐analysis of 352 patients from three placebo‐controlled trials (OT‐401, REVERSE, and CONFIRM) comparing outcomes in patients with HRS reported increased HRS reversal, 90‐day transplant‐free survival, and overall survival in patients with creatinine <3 mg/dl compared to those with higher creatinine values.^13^, ^17^, ^19^, ^21^ The benefit–risk of terlipressin use is unfavorable among patients with acute‐on‐chronic liver failure (ACLF) grade >3 or serum creatinine >5 mg/dl.^22^ Thus, early recognition and treatment of HRS is important to initiate terlipressin treatment. Previous studies have reported that patients with HRS who respond to terlipressin and albumin have reduced need for RRT after LT and lower odds of developing chronic kidney disease (CKD) 1 year after LT.^22^

Patients with HRS may be managed in hospital settings, which may differ based on teaching status and the presence of a liver transplant program. Our goal was to examine whether the survival of patients with HRS was different among these different hospital settings. Our hypothesis was that patients admitted to teaching transplant centers (TTCs) would have higher acuity of illness compared to non‐teaching hospitals (NTHs). We also hypothesized that overall and transplant‐free survival of patients with HRS would be higher at TTCs, partly due to the availability of LT and the presence of a multidisciplinary team comprising transplant hepatologists, transplant surgeons, nephrologists, as well as trainees.

## Methods

### Data source

The Nationwide Inpatient Sample (NIS) database, the largest inpatient healthcare database in the United States, was developed by the Agency for Healthcare Research and Quality.^23^ This database contains de‐identified hospitalization records of 35 million weighted hospitalizations annually. We included data for hospitalizations from 2016 to 2019. The database has been described in detail previously.^24^, ^25^, ^26^ Since the data obtained is publicly available de‐identified data, IRB approval was not required.

### Study population

**Figure 1:**
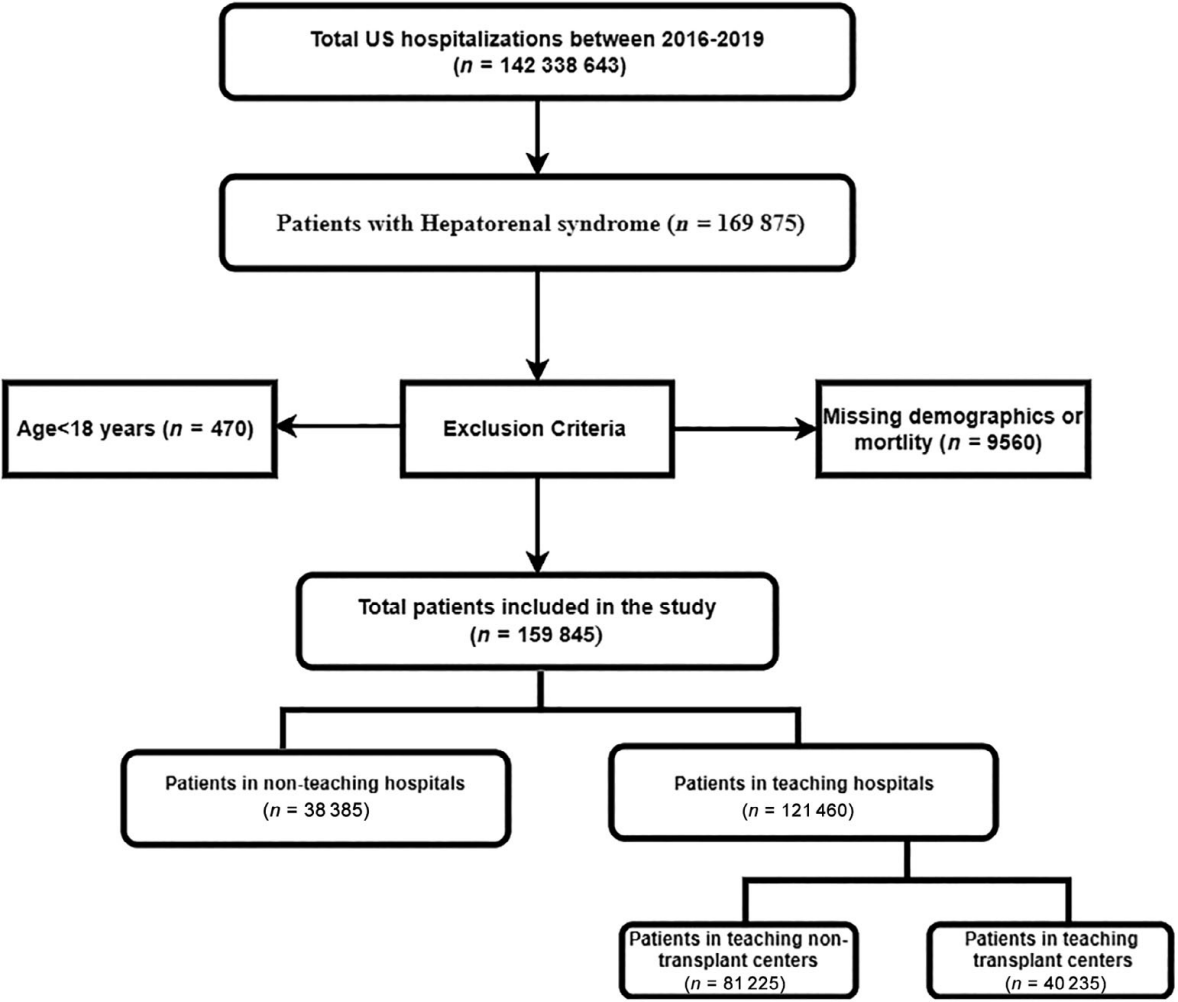
Flowchart of case selection for patients with hepatorenal syndrome.

Patients from the dataset were selected based on the International Classification of Diseases, Clinical Modification‐10th Revision (ICD‐10‐CM) codes. Patients hospitalized with primary or secondary discharge diagnoses of HRS were included in the study (ICD‐10‐CM codes K76.7). Patients with AKI without a diagnosis of HRS were excluded. Patients younger than 18 years and those with missing information on mortality and demographics were excluded. The study population is presented in Figure 1.

### Definition of teaching and transplant status of the hospital

According to Health Care Utilization Project (HCUP), which is the largest collection of longitudinal hospital care data, a hospital is considered a teaching hospital if it has one or more Accreditation Council for Graduate Medical Education (ACGME)‐approved residency programs, is a member of the Council of Teaching Hospitals (COTH), or has a ratio of full‐time equivalent interns and residents to beds of 0.25 or higher. A hospital was classified as a transplant center if it performed at least one documented LT in the year of admission. Based on this classification system, patients were initially divided into those receiving treatments in four types of hospitals, namely teaching transplant centers (TTCs), teaching non‐transplant centers (TNTCs), non‐teaching transplant centers (NTTCs), and non‐teaching non‐transplant centers. This approach has been used previously to stratify hospitals based on teaching and transplant status.^27^ Since there were only 45 LTs in NTHs, we decided to subclassify only THs into transplant centers (TCs) and non‐transplant centers (NTCs).

### Study variables

In addition to the teaching/transplant status of the hospital, other variables in our analysis included patient demographics (age group, gender, race, primary insurance, median income), hospital characteristics (region, bed size, and rural/urban location, prespecified by HCUP), and Elixhauser comorbidities.^28^ It is a well‐validated index based on ICD‐10‐CM codes meant to be used in large administrative data to predict mortality and hospital resource use. The index has 31 comorbidity categories. Furthermore, we obtained data on the etiology and decompensations of the disease and interventions (LT, RRT, paracentesis, transjugular intrahepatic portosystemic shunt [TIPS], mechanical ventilation, and pressor use).

### Study outcomes

The primary outcome was the relationship between the teaching/transplant status of the hospital and inpatient mortality, mortality among patients who received RRT, and transplant‐free survival in hospitalized patients with HRS.

### Statistical analysis

Descriptive statistics are presented in the table as the total number with percentages for categorical variables. The mean for continuous variables is reported for continuous measures. Baseline characteristics are compared using the Pearson chi‐square test for categorical variables and an independent *t*‐test for continuous variables. Univariate logistic regression was performed to study the effect of confounding variables on mortality. Only variables that met the cut‐off of *P* <0.1 were included in the multivariate analysis. We adjusted for patient demographics, hospital characteristics, liver disease etiology, liver disease decompensations, Elixhauser comorbidities, and interventions. A type I error of <0.05 was considered statistically significant. The data analysis was conducted using STATA 17.0 (Texas).

This study was conducted in compliance with the ethical standards of the responsible institution on human subjects as well as with the Helsinki Declaration.

## Results

Our study population included 159,845 patients. Of these, 38,385 (24.01%) were admitted to NTHs and 121,460 (75.99%) to THs. On further stratification of THs, 81,225 (50.81%) patients were admitted to TNTCs, while the remaining were admitted to TTCs (25.18%).

### Demographics and hospital characteristics

Most patients admitted with HRS were between 45 and 64 years of age (52.9%). A higher proportion of those admitted to TTCs were aged 45–64 years compared to TNTCs and NTHs (56.8% *vs* 51.7% and 51.2%, respectively). A lower proportion of patients aged >65 years was admitted to TTCs compared to TNTCs and NTHs (27.3% *vs* 38.6% and 35.8%, respectively). Most of the patients admitted were males (61%). The majority of the patients in the study were White (67.2%), followed by Hispanics (15.9%) and African Americans (9.8%). Significant differences were based on insurance status and median household income between the hospital groups. A higher proportion of Medicare insurance patients were in NTHs than in TNTCs and TTCs (46.4% *vs* 42.9% and 37.8%, respectively). All transplant centers were located in urban areas. On examining the transfer status, it was noted that 36.8% of the patients admitted to TTCs were transferred from another acute care facility. Detailed information is provided in Table 1.

**Table 1:** Patient demographics and hospital characteristics, classified by hospital's teaching and transplant status

Demographics	Non‐teaching hospitals, *n* (%)	Teaching non‐transplant, *n* (%)	Teaching transplant, *n* (%)	*P*‐value
Age category (years)				**<0.001**
18–44	3895 (10.2)	10 160 (12.5)	6425 (15.9)	
45–64	19 665 (51.2)	41 930 (51.7)	22 905 (56.8)	
>65	14 825 (38.6)	29 020 (35.8)	11 020 (27.3)	
Sex				**0.005**
Male	23 375 (60.9)	50 095 (61.8)	24 015 (59.5)	
Female	15 010 (39.1)	31 015 (38.2)	16 335 (40.5)	
Race				**<0.001**
White	27 260 (71)	52 720 (65)	27 435 (68)	
Black	2805 (7.3)	9065 (11.2)	3760 (9.3)	
Hispanic	5930 (15.5)	13 505 (16.7)	5980 (14.8)	
Asian/Pacific Islander	815 (2.1)	1810 (2.2)	915 (2.3)	
Primary expected payer				**<0.001**
Medicare	17 790 (46.4)	34 815 (42.9)	15 240 (37.8)	
Medicaid	8975 (23.4)	20 615 (25.4)	9360 (24.4)	
Private	8310 (21.7)	18 530 (22.9)	13 415 (25.2)	
Uninsured	1835 (4.8)	4415 (5.4)	1140 (2.8)	
Median household income				**<0.001**
Lowest quartile	13 180 (34.3)	25 395 (31.3)	11 585 (28.7)	
Second quartile	10 765 (28)	21 410 (26.4)	9940 (24.6)	
Third quartile	8395 (21.9)	19 530 (24.1)	10 220 (25.3)	
Highest quartile	6045 (15.8)	14 775 (18.2)	8605 (21.3)	
Hospital region				**<0.001**
Northeast	4405 (11.5)	15 630 (19.3)	7825 (19.4)	
Midwest	6555 (17)	16 130 (19.9)	10 185 (25.2)	
South	16 740 (43.6)	29 045 (35.8)	13 930 (34.5)	
West	10 685 (27.8)	20 305 (25)	8410 (20.8)	
Hospital location				**<0.001**
Rural	8770 (22.9)	0 (0)	0 (0)	
Urban	29 615 (77.2)	81 110 (100)	40 350 (100)	
Hospital bed size				**<0.001**
Small	4820 (12.6)	19 415 (23.9)	840 (2.1)	
Medium	11 175 (29.1)	27 510 (33.9)	4025 (10)	
Large	2390 (58.3)	34 185 (42.2)	35 485 (87.9)	
Transfer status				**<0.001**
Direct admission	34 785 (90.6)	69 765 (86)	23 590 (58.5)	
Transferred from acute care hospital	1870 (4.9)	7320 (9.3)	14 830 (36.8)	
Transferred in from another type of health facility	1575 (4.1)	3545 (4.4)	1740 (4.3)	
Transferred to different acute care hospital	4700 (12.2)	7360 (9.1)	1250 (3.1)	**<0.001**

### Etiology and complications of liver disease

Significant differences were noted among the patients admitted to different hospital settings based on the etiology and complications of liver disease. A higher proportion of patients in TTCs had hepatocellular carcinoma (HCC) compared to NTHs and TNTCs (9.5% *vs* 4.7% and 6.2%, respectively). There was a higher proportion of patients with non‐alcoholic steatohepatitis (NASH) in TTCs (19.84%), while a higher proportion of patients with alcohol‐related liver disease were admitted to TNTCs (52%). There was a higher incidence of ascites (78.9%), variceal bleeding (5.4%), and spontaneous bacterial peritonitis (13.7%) in TTCs compared to other hospital groups. The results of the etiology and decompensations of liver disease, stratified by teaching and transplant status, are presented in Table 2.

**Table 2:** Etiology and decompensations of liver disease, classified by the hospital's teaching and transplant status

	Non‐teaching hospitals, *n* (%)	Teaching non‐transplant, *n* (%)	Teaching transplant, *n* (%)	*P*‐value
Etiology				
Hepatitis B	600 (1.6)	1650 (2)	1030 (2.6)	**<0.001**
Alcoholic liver disease	18 570 (48.4)	42 200 (52)	19 465 (48.2)	**<0.001**
NASH	4560 (11.9)	9520 (11.7)	8005 (19.8)	**<0.001**
Hepatitis C	5175 (13.5)	12 020 (14.8)	6005 (14.9)	**0.03**
Hepatocellular cancer	1800 (4.7)	5025 (6.2)	3835 (9.5)	**<0.001**
Decompensations				
SBP	3750 (9.8)	9865 (12.2)	5520 (13.7)	**<0.001**
Variceal bleeding	1665 (4.3)	4245 (5.2)	2165 (5.4)	**0.007**
Hepatic encephalopathy	7030 (18.3)	15 440 (19)	5885 (14.6)	**<0.001**
Ascites	25 545 (66.6)	58 060 (71.6)	31 810 (78.8)	**<0.001**

Bold values signifies statistical significance.

NASH, non‐alcoholic steatohepatitis; SBP, spontaneous bacterial peritonitis.

### Patient comorbidities and interventions

The prevalence of comorbidities such as congestive heart failure, chronic valvular disease, diabetes, alcohol misuse, and drug abuse was lower in patients admitted to TTCs compared to other hospital groups. These results are presented in Table S1, Supporting information. A higher proportion of patients in TTCs underwent TIPS (0.9% *vs* 0.5% and 0.2%), paracentesis (55.8% *vs* 50% and 42.6%), RRT (30.7% *vs* 16.1% and 15.5%), and mechanical ventilation (24.6% *vs* 18.2% and 15.9%), compared to TNTCs and NTHs. Only 0.08% of liver transplants occurred at NTHs. The results are presented in Table 3.

**Table 3:** Interventions and severity of disease, stratified by hospital's teaching and transfer status

Interventions	Non‐teaching hospitals, *n* (%)	Teaching non‐transplant, *n* (%)	Teaching transplant, *n* (%)	*P*‐value
TIPS	95 (0.2)	375 (0.5)	380 (0.9)	**<0.001**
Paracentesis	40 570 (42.6)	22 525 (50)	79 455 (55.8)	**<0.001**
Renal replacement therapy	5955 (15.5)	13 080 (16.1)	12 370 (30.7)	**<0.001**
Liver transplantation	45 (0.1)	0 (0)	5325 (13.2)	**<0.001**
Mechanical ventilation	6095 (15.9)	14 790 (18.2)	9915 (24.6)	**<0.001**

TIPS, transjugular intrahepatic portosystemic shunt.

### Mortality and resource utilization

Mortality associated with a diagnosis of HRS was 26.15%. The mortality rate was the highest in TNTCs (24.4%), while the lowest rate was noted in TTCs (27.5%). After excluding patients who received a liver transplant, the mortality was noted to be highest in TTCs (27.41%), closely followed by TNTCs (27.32%). There were no significant differences in in‐hospital mortality among patients who received RRT between the three groups (*P* = 0.07). Patients admitted to TTCs had a longer length of stay and higher hospitalization charges than those admitted to NTHs and TNTCs. Information regarding mortality and resource utilization, stratified by teaching and transplant status, is presented in Table 4. After adjusting for confounding factors, admission to TTCs was independently associated with 25% lower odds of all‐cause mortality and transplant‐free mortality compared to NTHs (aOR = 0.76, 95% CI: 0.69–0.84, *P* < 0.001 and aOR = 0.75, 95% CI: 0.68–0.83, respectively). The results of multivariate logistic regression analysis are presented in Table 5. There was no statistically significant difference in the all‐cause and transplant‐free mortality between NTHs and TNTCs. There were no significant differences in in‐hospital mortality between the three groups among patients who received RRT.

**Table 4:** Mortality and resource utilization, stratified by teaching transplant centers

	Non‐teaching hospitals, *n* (%)	Teaching non‐transplant, *n* (%)	Teaching transplant, *n* (%)	*P*‐value
All‐cause mortality	9805 (25.5)	22 130 (27.3)	9865 (24.4)	**<0.001**
Transplant‐free mortality	9805 (25.5)	22 130 (27.3)	9630 (27.5)	**<0.001**
Mortality in patients who received RRT	1815 (30.5)	4455 (34.06)	3930 (31.8)	0.07
Length of stay	8.2 (±0.11)	9.6 (±0.08)	16 (±0.3)	**<0.001**
Total costs	$22 240.61 (±425.8)	$26 535.35 (±327)	$70 887.26 (±2835)	**<0.001**
Total hospitalization charges	$103 012.1 (±2427.8)	$113 179 (±1540.9)	$290 070.5 (±13 395)	**<0.001**

Bold values signifies statistical significance.

RRT, renal replacement therapy.

**Table 5:** Results of multivariate logistic regression model assessing the relationship between teaching/transplant status of the hospital and all‐cause/transplant‐free mortality

	Adjusted OR	95% Confidence interval	*P*‐value
	All‐cause mortality		
Non‐teaching Hospitals	Reference		
Teaching non‐transplant hospitals	1	0.92–1.08	0.98
Teaching transplant hospitals	0.75	0.68–0.84	**<0.001**
	Transplant‐free mortality		
Non‐teaching hospitals	Reference		
Teaching non‐transplant hospitals	1	0.93–1.08	0.97
Teaching transplant hospitals	0.75	0.67–0.83	**<0.001**
	Mortality in patients receiving RRT		
Non‐teaching hospitals	Reference		
Teaching non‐transplant hospitals	0.99	0.83–1.17	0.94
Teaching transplant hospitals	0.92	0.75–1.12	0.42

Bold values signifies statistical significance.

OR, odds ratio; RRT, renal replacement therapy.

## Discussion

Our study found that patients with HRS admitted to THs with a LT program had lower all‐cause mortality and transplant‐free mortality compared to those admitted to other hospital types. There were no significant differences in mortality between TNTCs and NTHs, despite the higher severity of the disease among patients admitted to THs without transplant programs.

We speculate that a major reason for improved survival in TTCs was accessibility to LT at TTCs. Approximately 13% of the patients admitted to TTCs with HRS received LT. The mortality rate in patients undergoing LT at TTCs was 4.4%, compared to 27.5% in patients who did not receive LT. Our results confirm previous reports, which have described the benefits of LT in patients with HRS.^7^, ^29^, ^30^, ^31^ Kaewput *et al*., in a study of 23,973 admissions with HRS, reported that LT was associated with a 67% lower mortality risk in HRS compared to those who did not receive LT.^8^ Most LTs occurred in THs (99.92%), while only a small number of them were performed in NTHs (0.08%).

To assess whether TTCs had better outcomes primarily due to the availability of LT, or whether additional factors were involved, we performed a separate analysis excluding patients who underwent LT. Our study noted 25% lower odds of in‐hospital mortality among patients who did not receive LT. Our findings suggest that patients with HRS have better outcomes when managed in a TTC, and a timely transfer of these patients from non‐transplant centers to TTCs could be beneficial in preventing worse outcomes among these patients.

Patients admitted to TTCs and TNTCs had higher severity of the disease than those at NTHs. A higher proportion of patients admitted to TTCs and TNTCs had evidence of liver disease complications such as ascites, variceal bleeding, HCC, and spontaneous bacterial peritonitis (SBP). TTCs and TNTCs also had a higher need for additional interventions such as TIPS, RRT, mechanical ventilation, and paracentesis than NTHs, suggesting higher severity. Furthermore, we noted that 37% and 9.3% of patients in TTCs and TNTCs, respectively, were transferred from another acute care facility. It has been previously documented that transferred patients have higher complications, mortality, and length of stay.^32^, ^33^

Interestingly, in our study, there were no significant differences in the mortality rates between the three hospital groups among patients who received RRT. The use of RRT is indicated in patients who are unresponsive to pharmacotherapy, develop volume overload or electrolyte derangements, or for those who are not candidates for LTs.^34^ Previous studies have reported that patients with HRS who require RRT have a dismal prognosis.^35^, ^36^ In a study by Tatum *et al*., the mortality among patients with HRS‐AKI, who received >7 days of continuous RRT, was reported to be 59%.^35^ Another study by McAllister *et al*., comparing patients receiving maintenance dialysis for HRS and acute tubular necrosis (ATN), reported a higher hazard of death and a lower hazard of recovery within 1 year among patients with HRS compared to ATN.^36^ These findings underscore the importance of early identification of HRS because once patients require RRT, mortality is higher.

We found that patients admitted to NTHs had a shorter length of stay and lower total hospitalization charges. However, this finding should be interpreted with caution because 12% of the patients admitted to NTHs were transferred to a different acute care hospital. By contrast, a longer length of stay and higher total hospitalization charges were noted in the TTCs. These differences could be due to greater disease complexity among patients admitted to TTCs. The use of multidisciplinary care teams, especially in potential LT candidates, may contribute to higher resource utilization at TTCs, but may be associated with better outcomes. A study by Zhang *et al*. of 307 patients with chronic liver disease patients reported 5‐year survival rates to be improved in patients under the care of a multidisciplinary team.^37^

A recent study by Singhal *et al*., using similar dataset from NIS, examined in‐hospital mortality of HRS among patients with NASH, alcoholic lever disease (ALD), and chronic viral hepatitis cirrhosis.^9^ Their study population had a similar mortality rate as in the current report (25.8%). Our study did not limit the patient population by diagnosis of cirrhosis or a specific liver disease etiology. Singhal *et al*. did not find differences in mortality based on the teaching status of the hospital. However, our study is different in that we examined differences in mortality not only based on the teaching status of the hospital but also based on the presence of a transplant program. Our findings of superior performance of TTCs compared to TNTCs and NTHs are similar to those of Bodek *et al*., who compared outcomes among patients admitted with hepatic encephalopathy.^27^

We acknowledge this study has limitations, which are largely due to the nature of data being obtained from a national database and the use of ICD‐10 codes for the diagnosis of HRS. We were also unable to obtain the time from onset of disease to identification of HRS, and relevant clinical data such as INR (International Normalized Ratio), creatinine, and sodium values were not available. As a result, we could not calculate MELD‐Na or Child–Pugh scores. We also did not have access to medications used to treat HRS, such as midodrine, albumin, octreotide, or vasopressin. Finally, the NIS only captures the hospitalization event, and thus patients cannot be tracked longitudinally. Nevertheless, the strengths of our study include the large database representing a national sample and confidence in the accuracy of hospital classification for the primary analyses in the study.

Our finding that patients admitted to TTCs have improved survival despite the higher severity of the disease has the potential to impact patient care. It is possible that early diagnosis of HRS and referral to a TTC may be associated with improved outcomes. In addition, increased education on early diagnosis and management of HRS focused on non‐transplant centers may also lead to early identification of patients, initiation of specific therapies, and prompt referral to an LT center among those who do not respond to these interventions. We hope the findings of this study may help guide future initiatives to improve management and survival for patients with this life‐threatening complication of end‐stage liver disease.

## Supporting information


**Table S1.** Patient comorbidities, classified by hospital's teaching and transplant status.

## Data Availability

The data is publicly available from HCUP national inpatient sample website https://hcup-us.ahrq.gov/nisoverview.jsp.
