# Supplementary material for: Outcomes among patients with hepatorenal syndrome based on hospital teaching and transplant status: Analysis of 159 845 hospitalizations
Source: JGH Open. 2023 Nov 27;7(12):848–54. doi: 10.1002/jgh3.12985 (PMC10757492; doi:10.1002/jgh3.12985)
Supplement: Supplementary file 1 — Table S1. Patient comorbidities, classified by hospital's teaching and transplant status. [file JGH3-7-848-s001.docx]

| **Comorbidities** | **Non-Teaching hospitals n(%)** | **Teaching Non-Transplant n(%)** | **Teaching Transplant n(%)** | **p-value** |
| --- | --- | --- | --- | --- |
| Congestive heart failure | 9,050 (23.6) | 18,180 (22.4) | 5,965 (14.8) | <0.001 |
| Cardiac arrhythmias | 8,155 (21.3) | 17,330 (21.4) | 8,300 (20.6) | 0.409 |
| Pulmonary circulation disorder | 2,175 (5.7) | 4,770 (5.9) | 3,025 (7.5) | <0.001 |
| Valvular Disease | 2,560 (6.7) | 5,295 (6.5) | 2,230 (5.6) | 0.006 |
| Hypertension, uncomplicated | 6,780 (17.7) | 14,530 (17.9) | 6,015 (14.9) | <0.001 |
| Paralysis | 175 (0.5) | 430 (0.5) | 235 (0.6) | 0.563 |
| Other neurological disorder | 8,140 (21.2) | 18,055 (22.3) | 7,060 (17.5) | <0.001 |
| Chronic pulmonary disease | 7,350 (19.2) | 13,775 (17) | 4,945 (12.3) | <0.001 |
| Diabetes, uncomplicated | 4,060 (10.6) | 6,965 (8.6) | 3,415 (8.5) | <0.001 |
| Diabetes, complicated | 9,590 (25) | 19,055 (23.5) | 8,740 (21.7) | <0.001 |
| Hypothyroidism | 4,800 (12.5) | 9,605 (11.8) | 4,555 (11.3) | 0.085 |
| Renal failure | 19,345 (50.4) | 38,030 (46.9) | 19,115 (47.4) | <0.001 |
| Peptic Ulcer Disease | 645 (1.7) | 1,595 (2) | 720 (1.8) | 0.273 |
| RA/ Collagen vascular disease | 705 (1.8) | 1,560 (1.9) | 820 (2) | 0.69 |
| Solid Tumor w/o metastasis | 4,650 (12.1) | 10,885 (13.4) | 5,485 (13.6) | 0.018 |
| AIDS/HIV | 115 (0.3) | 450 (0.6) | 150 (0.4) | 0.014 |
| Coagulopathy | 20,115 (52.4) | 46,450 (57.3) | 25,920 (64.2) | <0.001 |
| Metastatic Cancer | 2,690 (7) | 6,100 (7.5) | 1,960 (4.9) | <0.001 |
| Blood loss anaemia | 1,350 (3.52) | 2,645 (3.3) | 1,105 (2.7) | 0.035 |
| Defeciency anaemia | 3,360 (8.8) | 8,735 (10.8) | 3,835 (9.5) | <0.001 |
| Fluid and electrolyte disorder | 30,670 (79.9) | 65,880 (81.2) | 33,505 (83) | <0.001 |
| Weight loss | 9,400 (24.5) | 21,930 (27) | 16,610 (41.2) | <0.001 |
| Obesity | 5,610 (14.6) | 11,205 (13.8) | 5,905 (14.6) | 0.222 |
| Lymphoma | 275 (0.7) | 820 (1) | 395 (1) | 0.089 |
| Hypertension, complicated | 15,270 (39.8) | 30,755 (37.9) | 12,705 (31.5) | <0.001 |
| Depression | 4,330 (11.3) | 9,225 (11.4) | 5,535 (13.7) | <0.001 |
| Psychosis | 350 (0.9) | 755 (0.9) | 315 (0.7) | 0.496 |
| Drug abuse | 2,060 (5.4) | 4,490 (5.5) | 1,750 (4.3) | <0.001 |
| Alcoholic abuse | 19,715 (51.4) | 44,070 (54.3) | 19,830 (49.1) | <0.001 |
| Peripheral Vascular disorder | 1,945 (5.1) | 4,540 (5.6) | 1,440 (3.6) | <0.001 |

**Supplementary Table 1- Patient comorbidities, classified by hospital’s teaching and transplant status.**
